# Fast Microwave Synthesis of Hierarchical Porous Carbons from Waste Palm Boosted by Activated Carbons for Supercapacitors

**DOI:** 10.3390/nano9030405

**Published:** 2019-03-11

**Authors:** Chaozheng Liu, Weimin Chen, Shu Hong, Mingzhu Pan, Min Jiang, Qinglin Wu, Changtong Mei

**Affiliations:** 1 College of Materials Science and Engineering, Nanjing Forestry University, Nanjing 210037, China; lczwood@163.com (C.L.); cwmwood@163.com (W.C.); hongshu.320@163.com (S.H.); mzpan@njfu.edu.cn (M.P.); jmdmaster@126.com (M.J.); 2 Jiangsu Engineering Research Center of Fast-growing Trees and Agri-fiber Materials, Nanjing 210037, China; 3 School of Renewable Natural Resources, Louisiana State University, Baton Rouge, LA 70803, USA; QWu@agcenter.lsu.edu

**Keywords:** waste palm, microwave activation, specific surface area, porous carbon, supercapacitor

## Abstract

The synthesis of biomass-derived porous carbons (PCs) for supercapacitors by conventional two-steps method (chemical activation after carbonization) is complicated and time-consuming. In this study, we present a one-step microwave activation strategy to prepare hierarchically PCs from waste palm boosted by activated carbons (ACs). ACs with various specific surface areas (14, 642, and 1344 m^2^·g^−1^) were used for the first time to fast absorb microwave energy for converting waste palm into hierarchically PCs, that is, PC1, PC2, and PC3, respectively. The morphological and structural characterizations of PCs were studied. Also, the electrochemical performances of supercapacitors based on PCs as electrodes were further investigated. The results showed that the PC (PC1) boosted by AC with the lowest specific surface area possessed a porous structure (containing micro-, meso-, and macro- pores) with the largest specific surface area (1573 m^2^·g^−1^) and the highest micropore volume (0.573 cm^3^·g^−1^), as well as the suitable mesoporosity (29.69%). The as-prepared PC1 supercapacitor even in a gel electrolyte (PVA/LiCl) exhibited a high specific capacitance of 226.0 F·g^−1^ at 0.5 A·g^−1^ and presented excellent charge-discharge performance with an energy density of 72.3 Wh·kg^−1^ at a power density of 1.4 kW·kg^−1^ and 50.0 Wh·kg^−1^ at 28.8 kW·kg^−1^. Moreover, this promising method exhibited a simple, rapid, and cost-effective preparation of carbon materials from renewable biomass for energy storage applications.

## 1. Introduction

Driven by the increased concern about environment and energy issues, renewable materials-based energy storage devices are increasingly demanded [1,2,3]. Supercapacitors, as ideal devices for this purpose, have been known for their excellent cycling stability, rapid charge-discharge capability, and high power density [4,5]. Also, carbon materials have been proved as the most commonly used electrode materials for supercapacitors due to their chemical stability, porous structure, and low cost [6,7]. Generally, the hierarchical porous structure of the carbon material combines micro-, meso-, and macropores. Micropores (<2 nm) can be as the main storage sites to adsorb electrolyte ions, and mesopores (2–50 nm) can provide channels to facilitate the rapid diffusion of ions, while macropores (>50 nm) can shorten the ion transport paths [8,9]. Among numerous carbon materials, the hierarchical porous networks cannot only accelerate ion diffusion in pores [10,11] but also provide a mass of active sites to accommodate charge due to their high specific surface area [12,13]. In addition, three-dimensional (3D) structures of carbon materials as electrode materials can further facilitate the rapid diffusion of ions [14,15], and doping with heteroatoms (e.g., oxygen, nitrogen) can promote the wettability of carbon surface and participate in the Faradaic reaction contributing to pseudo-capacitance [16,17,18]. However, to fabricate this hierarchical porous carbon (PC) with heteroatom doping, hard templates (e.g., SiO_2_), complicated subsequent synthesis, and chemical agents (e.g., urea) are often required [19,20]. Consequently, the high cost and time-consuming synthesis have severely hindered the application of supercapacitors. Hence, it is essential to find a cost-effective and simple method to prepare this hierarchical PC for supercapacitor.

Currently, great attention has been drawn to use renewable biomass-derived PCs as promising electrode materials for supercapacitors [21,22]. Waste palm, an agricultural waste, herein is selected as a carbon precursor due to its low-cost, abundant sources, and high fixed carbon content [23]. However, the conventional synthesis methods (chemical activation after carbonization) for biomass-derived PCs usually require high energy consumption and a long time. Thus, it’s necessary to find an efficient and rapid approach to prepare sustainable and low-cost hierarchical PCs for supercapacitors. The microwave heating has many advantages over conventional heating, such as shorter duration, lower energy consumption, and higher heating efficiency [24,25]. Whereas potassium hydroxide which has been proved as an activator to enhance the porosity of carbon materials in high-temperature activation, mainly contributed to the formation of micropores [26,27]. Therefore, it is highly effective to prepare biomass-derived PCs for high-performance electric double layer capacitors by microwave-assisted KOH activation technique [28,29].

Some researchers found out that biomass is a poor microwave absorber, and the activated carbon (AC) could absorb microwave energy by itself, followed by the development of the pore structure [30,31]. In addition, biomass-based ACs by chemical and microwave activation has proven to be a promising approach for the synthesis of PCs. The microporous carbon from Pinewood-based AC was prepared by KOH and microwave heating for 30 min, and its specific surface area achieved 2044 m^2^·g^−1^ [32]. Moreover, the specific surface area of AC has a significant influence on its ability to absorb microwave energy [33]. Therefore, ACs with various specific areas from waste palm were used here for the first time to fast absorb microwave energy for converting waste palm into hierarchical PCs. Also, the ACs are directly activated into hierarchical PCs without any further processing.

In this study, we demonstrate a one-step approach to synthesize hierarchical PCs (i.e., PC1, PC2, and PC3 boosted with 14, 642, and 1344 m^2^·g^−1^ ACs, respectively) for supercapacitors from waste palm by microwave-assisted KOH activation in 5 min. To our knowledge, this novel method of utilizing ACs for boosting the rapid activation of waste palm has never been reported. The morphological and structural properties of PCs were studied using a series of characterizations, and the electrochemical properties of supercapacitors were further investigated. Benefiting from the interconnected hierarchical porous network and oxygen doping, the symmetric supercapacitor based on PC1 as electrodes exhibited a high specific capacitance and delivered considerable energy and power density. Moreover, this promising approach exhibited a simple, rapid, and cost-effective preparation of carbon materials from renewable biomass for energy storage applications.

## 2. Materials and Methods

### 2.1. Materials

Waste palm used in this study was obtained from United Palm Oil Mill, Nibong Tebal, Malaysia. Commercial palm shell-activated carbons were purchased from Mulinsen Activated Carbon Group (Nanjing, China). The polypropylene separator was offered by Yoteco New Energy Technology Co. Ltd. (Changzhou, China). All relevant chemical reagents, including potassium hydroxide (KOH), nickel foam (430 ± 30 g·m^−2^), acetylene black, ethyl alcohol, polytetrafluoroethylene (PTFE, 60 wt.%), hydrochloric acid (HCl), polyvinyl alcohol (PVA), and lithium chloride (LiCl), were obtained from Chemical Reagent Corporation (Nanjing, China).

### 2.2. Preparation of Porous Carbons

We chose three different specific surface areas of ACs (AC1, 14 m^2^·g^−1^; AC2, 642 m^2^·g^−1^; AC3, 1344 m^2^·g^−1^). The nitrogen sorption isotherms of all ACs are shown in Figure S1, and their specific surface area (S_BET_) and structure parameters are listed in Table S1. The waste palm samples were screened into a particle size of 20 mesh and then dried at 100 °C overnight. Subsequently, the dried samples were carbonized at 500 °C for 2 h at a heating rate of 10 °C·min^−1^ in a tube furnace under an N_2_ atmosphere, denoted as AC1. Two commercial ACs were screened into a particle size of 100 mesh and then dried at 100 °C for 12 h, denoted as AC2 and AC3. Waste palm, AC1, and KOH were mixed with a ratio of 1:1:6 by weight and the mixture was then heated in a microwave oven with the irradiation time of 5 min and power of 700 W. The collected sample was repeatedly washed with diluted HCl and distilled water to remove impurities and then dried at 100 °C for 12 h, denoted as PC1. For comparison, AC2/AC3 was substituted for AC1 to prepare the PCs, denoted as PC2/PC3. In addition, the waste palm was mixed with KOH at a ratio of 1:3 by weight without AC to prepare the PC, denoted as the control. However, we found that the control was only partially converted into the carbons, and so we didn’t discuss it. The field emission (FE)-SEM image of the control is shown in Figure S2, and it can be clearly seen that many impurities attached to its surface could not be washed out by acid.

### 2.3. Fabrication of Solid-State Symmetric Supercapacitors

**Figure 1:**
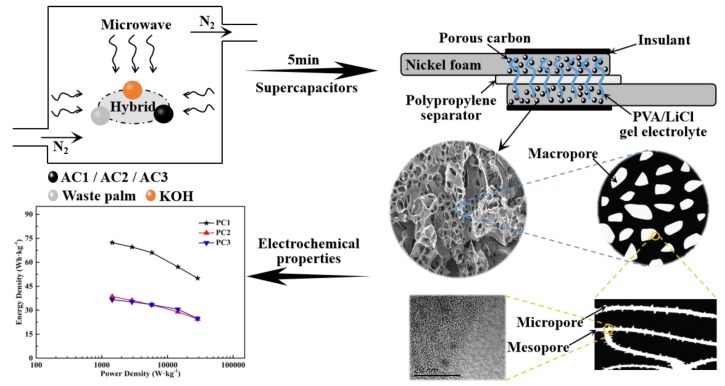
Schematic of the synthesis process for the solid-state symmetric supercapacitor. AC: activated carbon, PVA: polyvinyl alcohol.

80 wt.% PC, 10 wt.% acetylene black, 10 wt.% PTFE, and a certain amount of ethyl alcohol was well ground for 10 min to obtain a black paste. Then, the obtained paste was coated on half of the rectangular foamed nickel (20 mm × 10 mm) and dried at 100 °C for 8 h in a vacuum oven. Subsequently, the dried foamed nickel was pressed at 10 MPa for 1 min to obtain an electrode, and each electrode contained approximately 5.0 mg of PCs. The symmetric supercapacitor was fabricated using two identical electrodes, and the electrolyte was PVA/LiCl gel. The PVA/LiCl gel electrolyte was prepared by adding LiCl (21.2 g) and PVA (10 g) into deionized water (100 mL) at 85 °C for 1 h under constant stirring. Then, the two electrodes and the polypropylene separator were immersed in the gel electrolyte at ambient temperature for 6 h before assembly. Finally, the supercapacitor was assembled and then kept at ambient temperature for 12 h to remove excess water in the electrodes. The synthesis of the solid-state symmetric supercapacitor is schematically illustrated in Figure 1.

### 2.4. Characterizations and Measurements

The morphologies of the PCs were observed by a field-emission scanning electron microscope (FE-SEM, JSM-7600, JEOL, Tokyo, Japan) operated at 10.0 kV and a transmission electron microscope (TEM, JEM-2100, JEOL, Tokyo, Japan) with an accelerated voltage of 200 kV. The structures and compositions of the PCs were characterized via the following instruments: a Micromeritics ASAP 2020 instrument for N_2_ adsorption/desorption measurement, a Raman spectrometer (DXR532, Themor, Horiba Jobin Yvon, Paris, Franch) with a laser excitation wavelength of 532 nm, an X-ray diffraction spectrometer (XRD, D8 Advance, Bruker, Karlsruhe, Germany) with a range from 10° to 80° and a scan rate (2θ) of 3°/min, and an X-ray photoelectron spectra (XPS, AXIS UltraDLD, Thermo Fisher Scientific, Waltham, MA, USA) with the excitation source of Al Kα radiation at 1486.6 eV. The electrochemical measurements of the symmetric supercapacitor were assembled by two electrodes with exactly the same mass and were conducted in PVA/LiCl gel electrolyte. The Cyclic voltammetry (CV), galvanostatic charge/discharge measurements (GCD), and electrochemical impedance spectroscopy (EIS) were tested by an electrochemical workstation (Gamary Reference 600+). The CV tests were measured using different sweep speeds (5, 10, 20, 50, 100 mV·s^−1^) in the voltage range from −0.4 V to 0.4 V, and the GCD tests were investigated at different current densities (0.5, 1, 2, 5, 10 A·g^−1^). The EIS measurements were carried out in the frequency from 0.01 to 100 kHz. The specific capacitance (*C*, F·g^−1^), the energy density (*E*, Wh·kg^−1^), and the power density (*P*, kW·kg^−1^) of supercapacitor were evaluated by the following equations [34]:

(1) $$C=2I•\Delta t•{\left(m•\Delta V\right)}^{-1}$$

(2) $$E=0.5•C•\Delta V^{2}$$

(3) $$P=3.6•E•\Delta t^{-1}$$

where *I* is the discharge current (A), m is the mass (g) of PCs in a single electrode, and Δ*t* is the discharging time (s) corresponding to the voltage change Δ*V* (V).

## 3. Results and Discussion

### 3.1. Morphological and Structural Characterization

**Figure 2:**
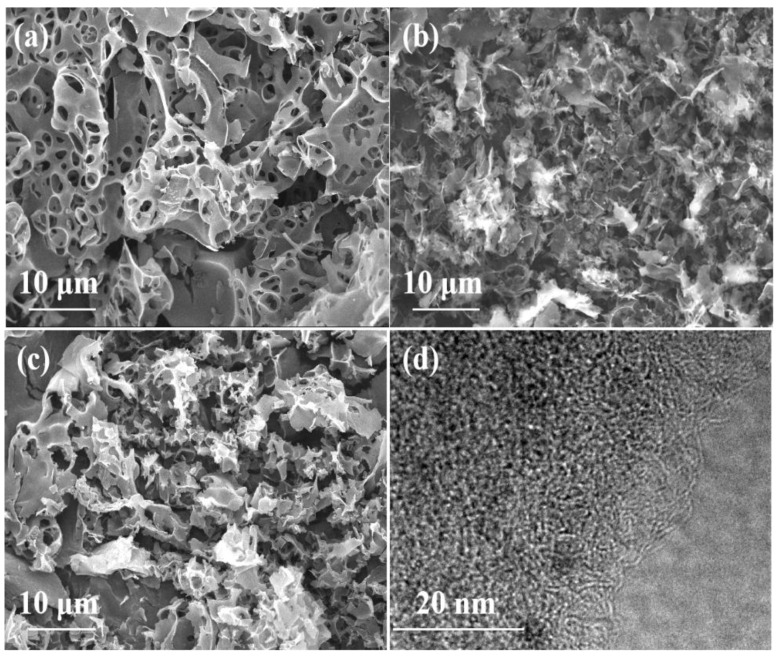
Field emission (FE)-SEM images of PC1 (**a**), PC2 (**b**), and PC3 (**c**) with low magnifications; TEM image of PC1 (**d**) with high magnifications. PC: porous carbon.

Figure 2a–c show the morphology images of the as-prepared samples by FE-SEM and directly prove the existence of macropores. As shown in the figure, the abundant pores in all PCs display irregular shapes and have the interconnected channels, showing a 3D distribution. This observation is due to the fact that KOH and carbon can react as following: 6KOH + 2C → 2K + 3H_2_ + 2K_2_CO_3_, to generate PCs by washing with HCl [35,36]. Also, the AC with the higher specific surface area could result in the more intense activation via absorbing more microwave energy, leading to the collapse of some macropores with large size (Figure 2b,c). Furthermore, Figure 2d shows the TEM images of PC1 with high magnification, and other samples are shown in Figure S3. All PCs exhibited the disordered and wormlike pore structure, demonstrating the existence of micropores and mesopores. As a result, the PC1 with hierarchical porous structure is apparently favorable for the rapid transportation of electrolyte ions.

**Figure 3:**
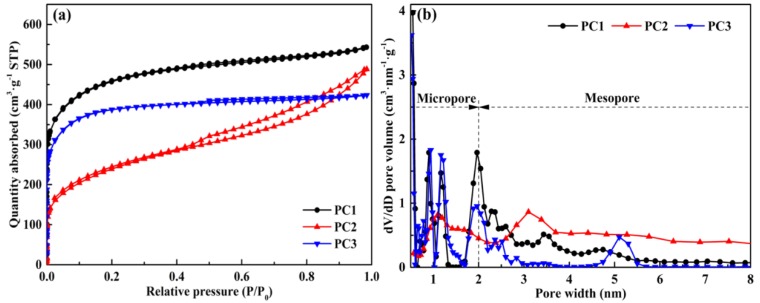
The pore structure of the porous carbons from waste palm (PC1, PC2, and PC3) of (**a**) N_2_ adsorption-desorption isotherms; (**b**) pore size distribution by density functional theory (DFT) method. PC: porous carbon.

The nitrogen sorption isotherms of all PCs (Figure 3a) are type IV in the IUPAC classification. The isotherms exhibit a sharp increase at a low relative pressure (<0.1 × P/P_0_), which indicates the formation of micropores [37]. Also, such isotherms show a hysteresis loop between the absorption and desorption branches at a high relative pressure (0.4 ~ 1.0 × P/P_0_), which is a characteristic for mesoporous materials [38,39,40], assigned to the aggregated carbon particles as demonstrated in Figure 2d. Figure 3b shows the pore size distribution of the PCs, which is based on the density functional theory (DFT) method. The results showed that most of the pore sizes in all samples were less than 2 nm, which confirm the high proportion of micropores in the texture. They also further revealed the formation of both micropores (<2 nm) and mesopores (2–50 nm) in all samples. The S_BET_ and structure parameters of the PCs are listed in Table 1. Compared with other samples, the PC1 possessed a porous structure with a larger specific surface area of 1573 m^2^·g^−1^ and a higher micropore volume of 0.573 cm^3^·g^−1^, as well as a suitable mesoporosity of 29.69%, corresponding to the maximum specific capacitance of the PC1 supercapacitor in Table 1. This result is due to the fact that the abundant micropores can boost the charge storage capacity, while mesopores can facilitate ion diffusion [29]. Combined with the morphological analysis, all the prepared PCs confirmed the presence of hierarchical porous structure (combine the micro-, meso-, and macropores).

**Figure 4:**
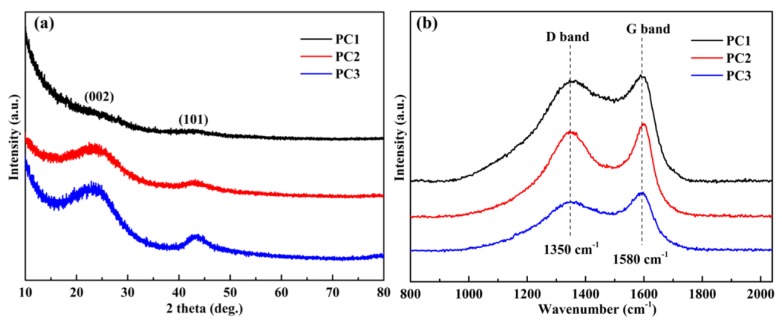
XRD patterns (**a**) and Raman spectra (**b**) of the porous carbons (PCs) samples.

The phase structure of PCs is also crucial to their performance as a supercapacitor. The XRD patterns of PCs (Figure 4a) exhibit two diffraction peaks at 23° and 43°, assigned to the characteristic diffraction peaks (002) and (101) of carbon materials [41]. It is noteworthy that the PC1 sample showed an obviously enhanced intensity in the small-angle region (2θ < 20°), demonstrating the existence of high-density micropores [42], thus corresponding to the results of the TEM analysis and the BET studies. In addition, Raman spectra (Figure 4b) was also measured to reflect the amorphous structure of PCs. It can be clearly seen that two characteristic bands were centered at 1350 cm^−1^ (D band) and 1580 cm^−1^ (G band), which respectively represents the disordered and ordered graphite structure of PCs [43]. The intensity ratio of D band to G band (I_D_/I_G_, Table 2) was used to evaluate the defect density of PCs. It can be observed that the I_D_/I_G_ ratios increased from 0.87 (PC3) to 0.98 (PC1) and 1.02 (PC2), indicating that there are many defect sites in the PC structure due to oxygen doping, agreeing well with the surface oxygen content of PCs in XPS analysis [44,45]. Such defects facilitate fast electrolyte ion diffusion into the PCs when used as an electrode material [46].

**Figure 5:**
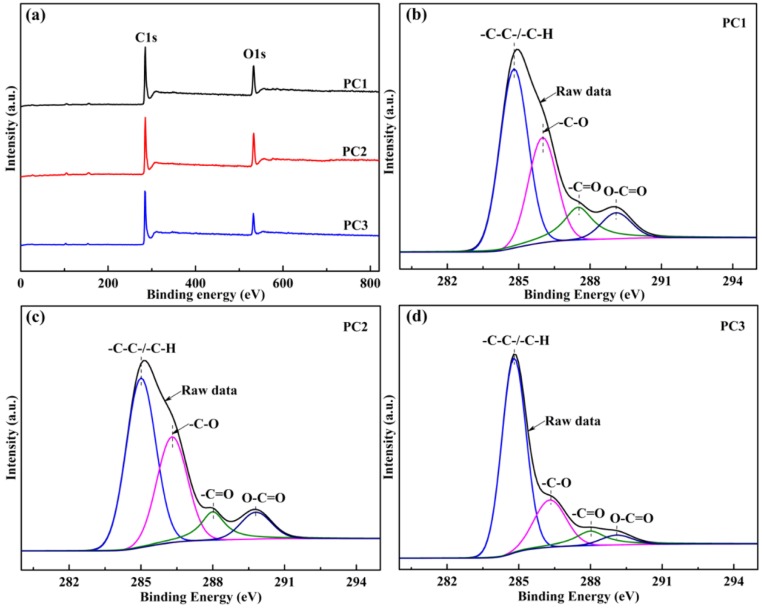
The X-ray photoelectron spectra (XPS) (**a–d**) of PCs: (**a**) the XPS wide-scan spectra, the high resolution for (**b–d**) C1s spectra of samples. PCs: porous carbons.

The surface chemical composition of the prepared PCs is measured by XPS, and the relative compositions of surface atomic elements and chemical groups in the C1s region of the PCs are shown in Table 2. The survey XPS spectra in Figure 5a clearly demonstrates the presences of C, N, and O elements. The oxygen-containing functional groups on the surface of PCs are studied by C1s spectra (Figure 5b–d) and O1s spectra (Figure S4). The high-resolution C1s spectrum, which is further deconvoluted into four carbon-related chemical groups (C1, -C-C- or -C-H at 284.8 eV; C2, -C-O at 286.3 eV; C3, -C=O at 288.0 eV; C4, O-C=O at 289.1 eV [47,48]), are presented. In addition, the O1s spectra reveals the existence of C=O quinine type group (O1, 530.7 ± 0.2 eV), carbon-oxygen double bond (O2, 531.9 ± 0.2 eV), carbon-oxygen ether-like single bond or carbon-oxygen single bond in hydroxyl groups (O3, 533.3 ± 0.2 eV) and chemisorbed oxygen (carboxylic groups) or water (O4, 534.2 ± 0.2 eV) [49,50,51]. It has been reported that oxygen doping is an effective method for improving the capacitive performance of supercapacitors, which can introduce extra pseudocapacitance and improve the electrolyte wettability and ion-accessible surface areas of the PCs [52,53]. The C1 relative content of PC1 is the lowest value by 48.76% in all samples, which indicates that the microwave activation of AC1 can destroy the carbon skeleton on the surface of PC and break the original C1. This result is due to the reaction of the oxygen-containing functional groups with the active sites on the surface of the PC, which is introduced to the PC surface to form stable oxygen-containing functional groups [54]. Moreover, this also explains the transition of C1 groups to C2, C3, and C4 on the surface of PC1 after microwave activation.

### 3.2. Electrochemical Performance

**Figure 6:**
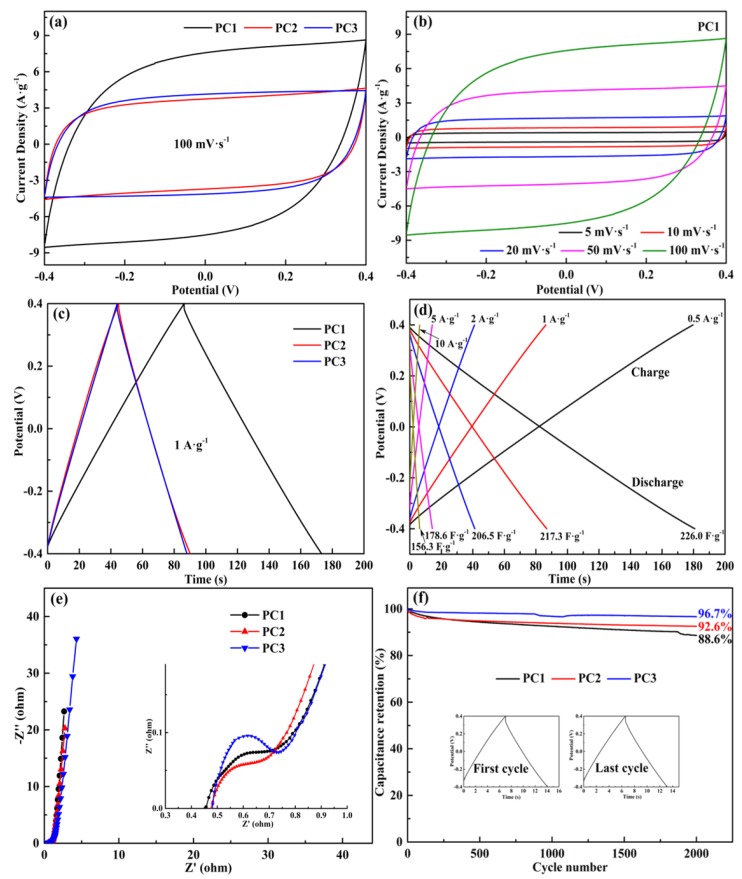
(**a**) The cyclic voltammetry (CV) curves of the PC1, PC2, and PC3 supercapacitors at a scan rate of 100 mV·s^−1^; (**b**) The CV curves of the PC1 supercapacitor at different scan rates; (**c**) The galvanostatic charge/discharge (GCD) curves of the PC1, PC2, and PC3 supercapacitors at a current density of 1 A·g^−1^; (**d**) The GCD curves of the PC1 supercapacitor at different current densities; (**e**) The electrochemical impedance spectra of the PC1, PC2, and PC3 supercapacitors; (**f**) The cycle life of the PC1, PC2, and PC3 supercapacitors at a current density of 5 A·g^−1^ for 2000 cycles. PC: porous carbon.

We fabricated the symmetric supercapacitors using PCs as the electrodes and tested their capacitive performance. Figure 6a compares the CV curves of the PC1, PC2, and PC3 supercapacitors at a scan rate of 100 mV·s^−1^. The quasi-rectangular CV shape and the dramatically enhanced current response indicate that supercapacitors can store much energy through a rapid ion adsorption mechanism, which reflects a good rate performance [55,56]. This result is due to the hierarchical porous structures of the PCs, as indicated by the morphological and structural analysis, and the promotion of good permeation and transportation of electrolyte ion from mesopores to micropores [57]. Figure 6b shows the CV curves of the PC1 supercapacitor at different scan rates in the range of 5–100 mV·s^−1^, and the other two supercapacitors are shown in Figure S5a,b. The rectangular CV shape of the PC1 supercapacitor at different scan rates suggests that the PC1 is an ideal electrode material for the symmetric supercapacitor.

Figure 6c shows the GCD curves of the PCs supercapacitors measured at a current density of 1 A·g^−1^. The symmetric and quasi-triangular shape was exhibited in all the GCD curves, demonstrating the excellent coulombic efficiency and electrochemical reversibility of all supercapacitors [58]. Also, the specific capacitances of the PC1, PC2, and PC3 supercapacitors calculated at a current density of 1 A·g^−1^ were 217.3 F·g^−1^, 112.7 F·g^−1^, and 110.1 F·g^−1^, respectively. Figure 6d shows the GCD curves of the PC1 supercapacitor calculated at different current densities ranging from 0.5 to 10 A·g^−1^ and its specific capacitance. The specific capacitance of the PC1 supercapacitor decreased with the rapid increase of the current density, which is due to the low ion-accessibility of micropores at high current density [9]. The specific capacitance of the PC1 supercapacitor reached 226.0 F·g^−1^ at a current density of 0.5 A·g^−1^ and remained 156.3 F·g^−1^ at a large current density of 10 A·g^−1^ even in a gel electrolyte (PVA/LiCl), showing excellent rate capability (69.16%). As shown in Figure S5c, the specific capacitances of the PC2 and PC3 supercapacitors at different discharge current density are calculated from the GCD curves. In consequence, the large specific surface area, the high micropore volume, as well as the suitable mesoporosity, were considered to be responsible for the high specific capacitance of PC1 supercapacitor.

Figure 6e shows the Nyquist plots of all the three supercapacitors with inset showing the high-frequency region. All the supercapacitors exhibited low ESR values (0.43–0.47Ω), which is mainly attributed to the high content of oxygen heteroatom and hierarchical porous structure in PCs [57]. Moreover, it has been proved that electrolyte ions can be transported and diffused rapidly in PCs. All the curves deviated from *Y*-axis in the low-frequency region, which indicates that the supercapacitor capacitance is derived not only from the double layer capacitance but also from the pseudo-capacitance [18]. Especially, the PC1 supercapacitor showed the highest double layer capacitance performance in all supercapacitors and its curve was closer to the *Y*-axis than others, which is due to the well-developed pore structure. In the intermediate frequency region, all supercapacitors exhibited a short Warburg region, which demonstrates that the electrolyte ion diffusion path is relatively short and thus the electrolyte ions can reach the surface of the PC easily [6]. The cycle life of the PC1, PC2, and PC3 supercapacitors at a current density of 5 A·g^−1^ for 2000 cycles are shown in Figure 6f. The PC1 supercapacitor showed good stability, and the specific capacitance was maintained 88.6% after 2000 cycles, which is due to the interconnected porous structure of the PCs. The specific capacitances of all supercapacitors were reduced after 2000 cycles, which is attributed to the irreversible reaction of the surface oxygen functional groups [59].

**Figure 7:**
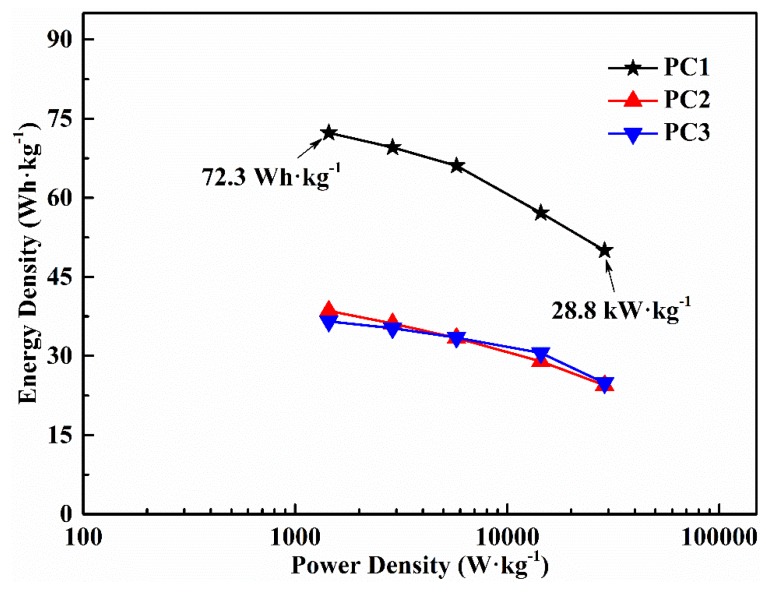
Ragone plot of porous carbons (PCs)-based symmetric supercapacitors.

Ragone plot (Figure 7) summarizes the energy density and power density, which evaluates the energy-power output properties of the solid-state symmetric supercapacitors. Figure S5d shows the relationship between the specific capacitance, the energy density, and the power density of the PC1 supercapacitor. The symmetric supercapacitor based on the PC1 in a gel electrolyte (PVA/LiCl) delivered a high energy density of 72.3 Wh·kg^−1^ at a power density of 1.4 kW·kg^−1^ and maintained an energy density of 50.0 Wh·kg^−1^ at a high power density of 28.8 kW·kg^−1^. It was noted that the power density of PC1 could vary in a wide range while its energy density was not obviously affected, demonstrating the excellent rate capability. These results indicate that the PCs from waste palm boosted by ACs can be used as active materials to prepare low cost and high-performance supercapacitors.

## 4. Conclusions

In summary, we have successfully developed a simple, rapid, and cost-effective one-step microwave activation strategy to utilize ACs to fast absorb microwave energy for converting waste palm into hierarchical PCs for supercapacitors. Results showed that the PC1 possessed a porous structure with the largest specific surface area (1573 m^2^·g^−1^) and the highest micropore volume (0.573 cm^3^·g^−1^), as well as the suitable mesoporosity (29.69%). The obtained PC1 supercapacitor even in an electrolyte gel (PVA/LiCl) exhibited a high specific capacitance of 226.0 F·g^−1^ at 0.5 A·g^−1^ and remained 69.16% at 10 A·g^−1^, indicating excellent rate capability. It also demonstrated a high energy density of 72.3 Wh·kg^−1^ at a power density of 1.4 kW·kg^−1^ and maintained an energy density of 50.0 Wh·kg^−1^ at a high power density of 28.8 kW·kg^−1^, as well as a good cycling performance (88.6% of the capacitance retention after 2000 cycles). Moreover, we hope this approach can open up a new way to prepare renewable biomass-derived carbon materials for advanced energy storage applications in the future.

## Figures and Tables

**Table 1:** Structure parameters of carbon samples and their capacitive performance.

Sample	S_BET_ (m^2^·g^−1^)	V_t_^a^ (V_mic_^a^) (cm^3^·g^−1^)	Mesoporosity (%)	Specific Capacitance ^b^ (F·g^−1^)	Capacitance Retention (%)
PC1	1573	0.815(0.573)	29.69	226.0	88.6
PC2	822	0.717(0.295)	58.86	120.5	92.6
PC3	1349	0.635(0.550)	13.39	114.3	96.7

^a^ Total pore volume (V_t_) and micropore volume (V_mic_) calculated by density functional theory (DFT) method. ^b^ Specific capacitance of the PCs supercapacitors calculated by galvanostatic charge/discharge (GCD) testing at a current density of 0.5 A·g^−1^. PC: porous carbon.

**Table 2:** The relative compositions of surface atomic elements and chemical groups, and I_D_/I_G_ values of the porous carbon samples (PC1, PC2, PC3).

Samples	I_D_/I_G_	Surface Atomic Elements (%)			Chemical Groups in C1s Region (%)			
		C	N	O	-C-C- or -C-H	-C-O	-C=O	O-C=O
PC1	0.98	82.31	0.13	17.56	48.76	29.06	14.25	7.93
PC2	1.02	80.54	0.15	19.31	49.51	31.99	10.11	8.39
PC3	0.87	86.35	0.96	12.69	65.91	20.92	8.92	4.25

## Supplementary Materials

The Supplementary Materials are available online at https://www.mdpi.com/2079-4991/9/3/405/s1.
