# Supplementary material for: Fast Microwave Synthesis of Hierarchical Porous Carbons from Waste Palm Boosted by Activated Carbons for Supercapacitors
Source: Nanomaterials (Basel). 2019 Mar 11;9(3):405. doi: 10.3390/nano9030405 (PMC6473988; doi:10.3390/nano9030405)
Supplement: Supplementary file 1 [file nanomaterials-09-00405-s001.pdf]

Supplementary Material

Fast Microwave Synthesis of Hierarchical Porous Carbons from Waste Palm Boosted by Activated Carbons for Supercapacitors

Chaozheng Liu[1,2], Weimin Chen[1,2], Shu Hong[1,2], Mingzhu Pan[1,2], Min Jiang[1,2], Qinglin Wu[3] and Changtong Mei[1,2*]

[1]College of Materials Science and Engineering, Nanjing Forestry University, Nanjing 210037, China
[2]Jiangsu Engineering Research Center of Fast-growing Trees and Agri-fiber Materials, Nanjing 210037, China
[3]School of Renewable Natural Resources, Louisiana State University, Baton Rouge, LA 70803, USA

*Corresponding author:

Tel.: +86 025 85427742

E-mail address: mei@njfu.edu.cn (CT. Mei).

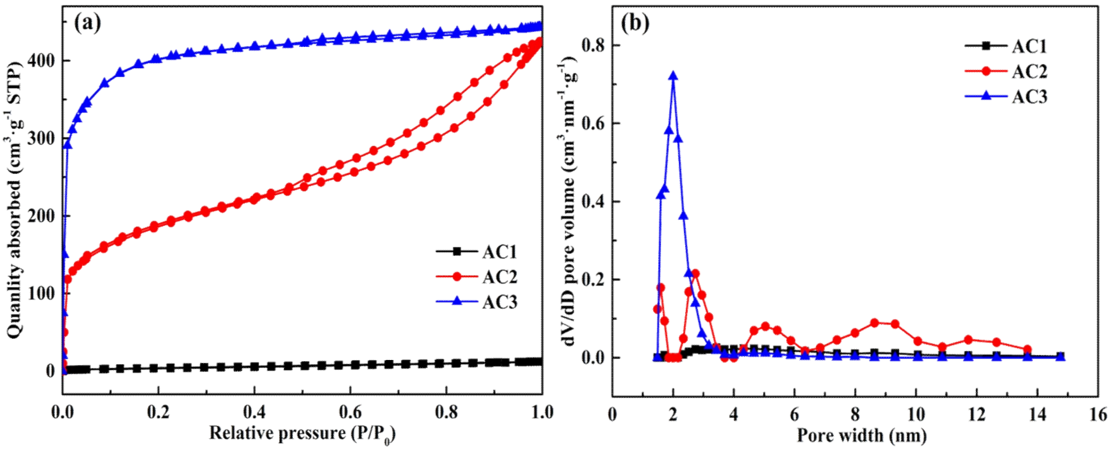

Figure S1. The Pore structure of the biochars (AC1, AC2, and AC3) of (**a**) N_2 adsorption-desorption isotherms, (**b**) The pore size distribution by density functional theory (DFT) method. AC: activated carbon.

Table S1. Structure parameters of activated carbons (AC1, AC2, and AC3).

Samples	S_{BET} (m²/g)	V_t[a] (cm³/g)	V_{mic}[a] (cm³/g)	Microporosity (%)
AC1	14	0.012	0.0002	1.7
AC2	642	0.173	0.122	70.5
AC3	1344	0.546	0.497	91.0

[a]Total pore volume (V_t) and micropore volume (V_{mic}) calculated by density functional theory (DFT) method. AC: activated carbon.

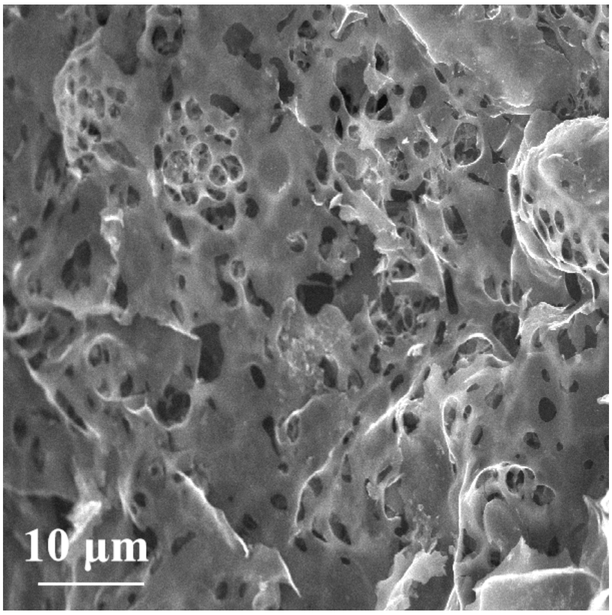

Figure S2. The field emission (FE)-SEM images of the control with low magnification.

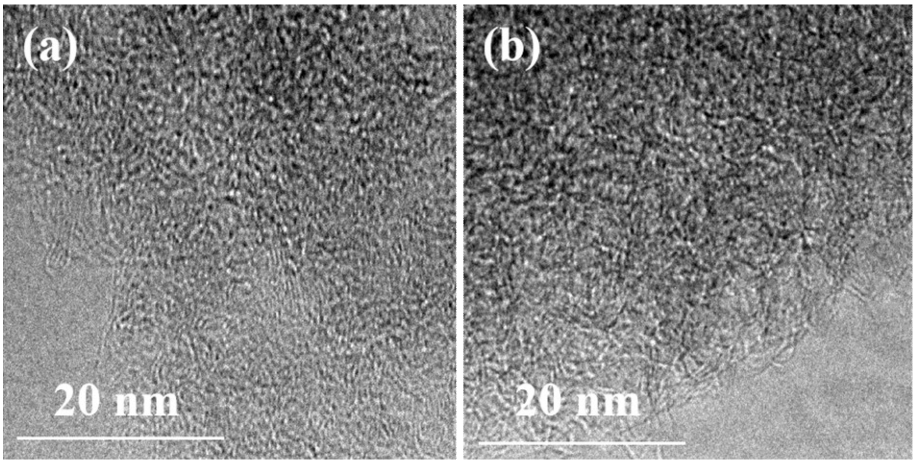

Figure S3. TEM images of PC2 (**a**) and PC3 (**b**) with high magnification. PC: porous carbon.

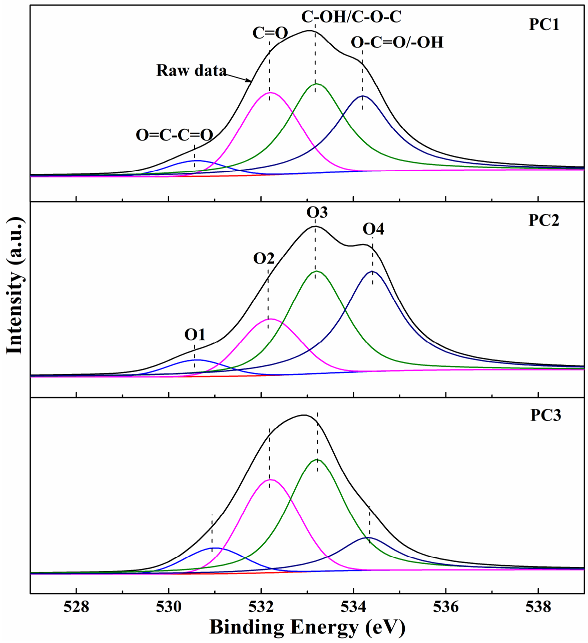

Figure S4. The high resolution for O1s X-ray photoelectron spectra (XPS) of PCs. PC: porous carbon.

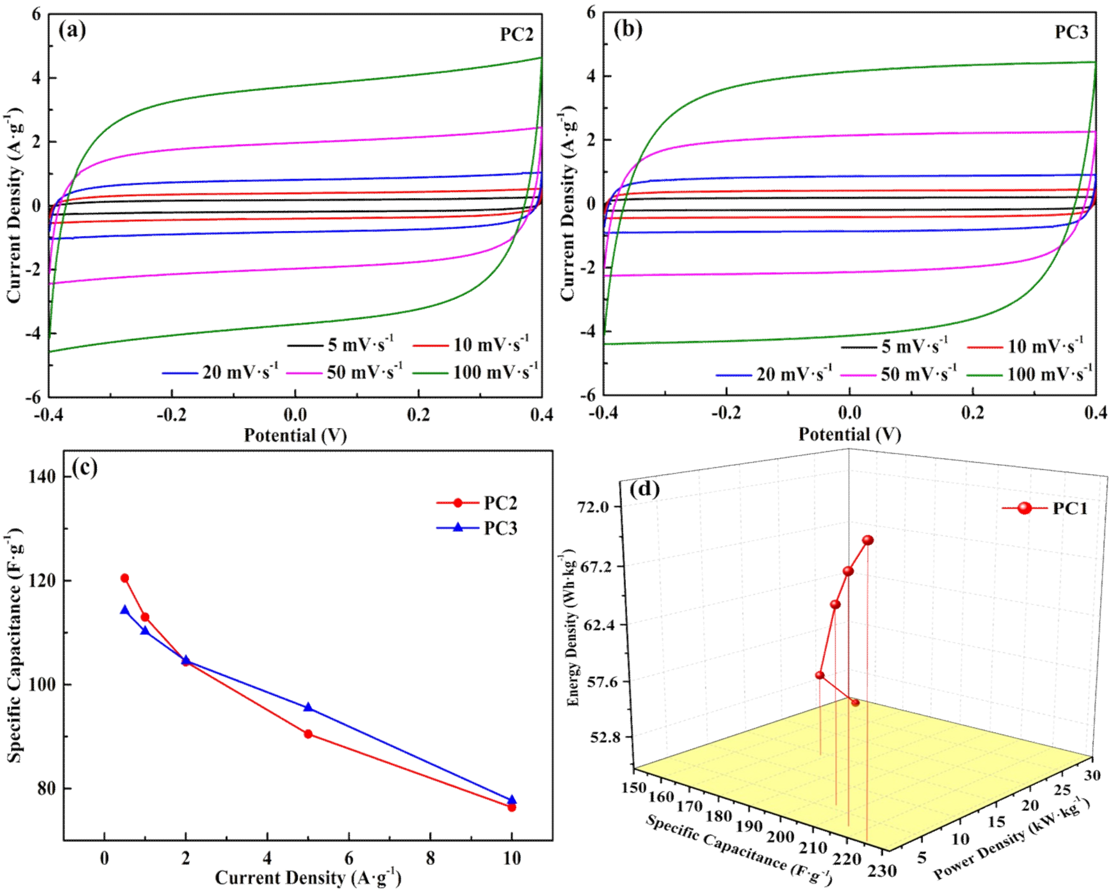

Figure S5. The cyclic voltammetry (CV) curves of the PC2 (**a**) and PC3 (**b**) samples at different scan rates, and (**c**) the specific capacitances of the PC2 and PC3 samples calculated from the galvanostatic charge/discharge (GCD) curves at different discharge current density, as well as (**d**) the 3D profile of specific capacitance-energy density-power density of PC1. PC: porous carbon.
